# Creation of a Plant Metabolite Spectral Library for Untargeted and Targeted Metabolomics

**DOI:** 10.3390/ijms24032249

**Published:** 2023-01-23

**Authors:** Yangyang Li, Wei Zhu, Qingyuan Xiang, Jeongim Kim, Craig Dufresne, Yufeng Liu, Tianlai Li, Sixue Chen

**Affiliations:** 1 College of Horticulture, Shenyang Agricultural University, Shenyang 110866, China; 2 Department of Biology, Genetics Institute, University of Florida, Gainesville, FL 32611, USA; 3 Horticultural Sciences Department, University of Florida, Gainesville, FL 32610, USA; 4 Thermo Scientific Training Institute, West Palm Beach, FL 32407, USA; 5 Department of Biology, University of Mississippi, Oxford, MS 38677, USA

**Keywords:** metabolomics, spectral library, mzVault, pseudo-targeted method, Arabidopsis

## Abstract

Large-scale high throughput metabolomic technologies are indispensable components of systems biology in terms of discovering and defining the metabolite parts of the system. However, the lack of a plant metabolite spectral library limits the metabolite identification of plant metabolomic studies. Here, we have created a plant metabolite spectral library using 544 authentic standards, which increased the efficiency of identification for untargeted metabolomic studies. The process of creating the spectral library was described, and the mzVault library was deposited in the public repository for free download. Furthermore, based on the spectral library, we describe a process of creating a pseudo-targeted method, which was applied to a proof-of-concept study of Arabidopsis leaf extracts. As authentic standards become available, more metabolite spectra can be easily incorporated into the spectral library to improve the mzVault package.

## 1. Introduction

In the post-genomics era, metabolomics is an indispensable system biology tool for understanding almost all the biological processes that involve signal transduction and metabolism [1]. Currently, the technologies and tools employed in metabolomic studies include non-destructive nuclear magnetic resonance spectroscopy (NMR) and mass spectrometry (MS)-based methods, e.g., gas chromatograph-MS and liquid chromatography (LC)-MS [2,3]. Among them, high-resolution MS coupled with LC is the most widely utilized technology for plant metabolomics [4]. For example, LC-MS-based metabolomics has been used toward understanding the mechanisms of plant stress responses through profiling cellular metabolite changes in the drought stress of trifoliate orange [5], the salt stress of broccoli [6], temperature stress of Arabidopsis [7], ultraviolet stress of *Mahonia bealei* [8], and oxidative stress of rice [9], just to name a few.

There are two major approaches for MS-based metabolomics: targeted and untargeted metabolomics [10]. Typically, the targeted metabolomics involved identifying and quantifying a group of known metabolites using selected reaction monitoring (SRM or SRMs when multiple transitions are monitored) with tandem mass spectrometers [11]. However, the targeted method is often limited to profiling the metabolites with available authentic standards. By contrast, untargeted metabolomics strives to detect, identify, and quantify as many metabolites as possible in a single or integrated analysis without authentic standards or prior knowledge of annotated metabolites [12]. The identification of metabolite features (including LC retention time, MS^1^, and MS^n^) from untargeted metabolomics is largely dependent on searching the MS/MS or MS^n^ spectra against existing databases such as MassBank [13], METLIN [14], Global Natural Product Social Molecular Networking (GNPS) [15], mzCloud, Human Metabolome Database (HMDB) [16], and Spektraris [17]. Only a limited number of databases, such as mzCloud and MassBank, have reported MS^n^ spectra in the above MS/MS databases, but they contain mixtures of mostly animal/human-related metabolites and some plant metabolites. These databases lack the benefits of community contribution and data curation [18,19]. Furthermore, there are many overlapping compounds in mzCloud or MassBank, and most of the same compounds have redundant names. It is difficult and time-consuming to distinguish the bona-fide plant metabolites from spectral-matched compounds. Therefore, the creation of a plant metabolite spectral library will greatly benefit the chemical annotation of plant metabolites in metabolomic studies.

In the present study, we generated a plant spectral library under different collision energies of 544 authentic compounds that are produced in Arabidopsis. The mass spectra form a mzVault library, which is available to the community for use, further improvement and growth. The utility of the spectral library was tested in untargeted and targeted metabolomic applications using Arabidopsis seedlings. It is an important resource for scientists conducting plant metabolomics and for the larger biology community.

## 2. Materials and Methods

### 2.1. Authentic Compounds and Plant Materials

Authentic compounds were purchased from Sigma Aldrich (St. Louis, MO, USA) and were dissolved at a final concentration of 1 ng/µL in water (Supplemental Table S1). Compounds that did not dissolve well in water were dissolved in 75% methanol as an alternative solvent. Arabidopsis melatonin triple mutant, *snat1asmt1comt1* was generated by crossing *snat1* (salk_020577) [20], *asmt1* (salk_067718) [20], and *comt1* (CS25167) [21]. *Arabidopsis thaliana* seeds Columbia (Col-0) wild-type (WT) and a melatonin triple mutant were surface-sterilized using 30% bleach for 10 min and were germinated on a half-strength Murashige and Skoog (MS) medium after a 4 °C treatment in the dark for 2 days [22]. After growing on the MS medium for 10 days, the seedlings were transferred to soil in a growth chamber under a photosynthetic flux of 160 μmol photons m^−2^ sec^−1^ and an 8 h light/16 h dark cycle for 6 weeks until the collection of leaves for metabolomic analyses.

### 2.2. Acquisition of Metabolite MS1 and MS2 Spectra

Authentic compounds were injected one at a time onto an Accucore™ C18, 2.6 μm 2.1 × 30 mm column (PN#17126-032130, Thermo Fisher Scientific, Vilnius, Lithuania) at a flow rate of 400 μL/min using a Thermo Scientific Vanquish^TM^ Horizon UHPLC system (San Jose, CA, USA). The injection volume was 10 μL. The run was 15 min in total, and the experiment was composed of a linear gradient from 0.1% formic acid and 10 mM ammonium formate in water to 0.1% formic acid and 10 mM ammonium formate in acetonitrile at 35 °C. The Orbitrap Q Exactive^TM^ (Thermo Scientific, San Jose, CA, USA) was run at 70,000 resolution (*m/z* 200) for MS1 with positive and negative switching and data-dependent MS/MS at a 17,500 resolution in each mode. The compounds with the expected precursor ion and MS2 fragment information (with signal-to-noise ratios of >10) were retained. For the compounds with fewer than three MS2 fragments, repeated injections with multiple collision energies were conducted to obtain additional MS2 fragments. The fragmentation was performed using different normalized collision energy (NCE) settings (10, 15, 20, 30, 35, 40, 50, 60, 70, 80, 90, and 120 eV). The compounds that failed to produce satisfactory MS2 spectra were removed from the library list.

### 2.3. mzVault Spectral Library Construction

The positive and negative MS1 and MS2 spectra of the authentic compounds were obtained using the data-dependent acquisition of the Vanquish Q Exactive Orbitrap LC-MS/MS system (Thermo Scientific, San Jose, CA, USA). Authentic compounds which did not yield MS2 spectra with at least three fragment ions were reinjected with targeted parallel reaction monitoring (PRM) on the Q Exactive system. An inclusion list of precursor m/z and charge states for targeted MS/MS, an automatic gain control (AGC) of 5 × 10^5^ [23], and a maximum injection time (IT) of 55 ms were used. The NCE settings were evaluated at 10, 15, 20, 30, 35,40, 50, 60, 70, 80, 90, and 120 eV. Each raw mass spectrum was filtered and recalibrated based on theoretical accurate mass, resulting in recalibrated spectral trees in the mzValult. The best spectra or a few spectra when the best was not clear were chosen for the library. The PRM quantitation ion and, ideally, three confirming ions of metabolites were added to the mzVault. Optimized collision energies on a TSQ Quantis^TM^ triple quadrupole MS (Thermo Scientific, San Jose, CA, USA) were derived, and all the metabolites were added to a TraceFinder^TM^ compound database. All the metabolites in the compound database were mixed at 1 ng/µL concentrations and used an unscheduled selected reaction monitoring (SRM) method to measure chromatography performance on a HILIC column, including Accucore^TM^ 150 Amide (16726-152130, 2.1 × 150 mm, 2.6 μm, 0.1% FA, 10 mM AmmForm), a reverse phase Acclaim™ Polar Advantage II (063187, 2.1 × 150 mm, 3 μm, 0.3% Heptafluorobutyric Acid or 0.1% Difluoroacetic Acid), and a HILIC-IEX Acclaim™ Trinity P2 (085432, 2.1 × 100 mm, 3 μm, pH Gradient) (Thermo Scientific, San Jose, CA, USA).

### 2.4. Metabolite Extraction of Leaves from Arabidopsis Plants

The extraction of metabolites was performed according to the method of Fiehn et al. with minor modifications [24]. Briefly, lyophilized Arabidopsis leaf samples (10 mg dry weight) were used, and a total of five biological replicates were conducted. Prior to extraction, an internal standard mixture (10 μM each of lidocaine and 10-camphorsulfonic acid) was added to each sample. After adding 1 mL of the extraction solvent I (acetonitrile: isopropanol: water, 3:3:2), the samples were vortexed for 15 min at 4 °C, sonicated for 15 min in ice-water, and centrifuged at 13,000× *g*, 4 °C for 15 min. They were sequentially extracted with extraction solvent II (acetonitrile: water, 1:1) and extraction solvent III (80% methanol). The supernatants were combined, lyophilized, and reconstituted in 100 μL 30% methanol with 0.1% formic acid.

### 2.5. Untargeted Metabolomics of Leaves of Arabidopsis Plants

Untargeted metabolomic data were generated from the data dependent MS/MS acquisition on the Q-Exactive mass spectrometer. The samples were injected onto the Vanquish Horizon UHPLC system, which runs at a flow rate of 400 μL/min. A 30 min run composed of a linear gradient from 0.1% formic acid and 10 mM ammonium formate in water to 0.1% formic acid and 10 mM ammonium formate was conducted in acetonitrile at 35 °C. The Q Exactive MS was run at 70,000 resolution (*m/z* 200) for MS1 with positive and negative switching and data dependent MS/MS at a 17,500 resolution in each mode. Ion fragmentation was induced by HCD, with a default charge state of 1. Full MS1 used one microscan, an AGC target of 1x10^6^, and a scan range from 200 to 2000 *m/z*. The ddMS2 scan used one microscan, an AGC target of 5x10^5^, max IT of 46 ms, a loop count of 3, and an isolation window of 1.3 *m/z*. For untargeted metabolomics data analysis, Compound Discoverer^TM^ 3.0 software (Thermo Fisher Scientific, San Jose, CA, USA) was used [25]. The mzCloud and mzVault databases were used for metabolite identification with a mass tolerance of 5 ppm.

### 2.6. Targeted Metabolomics of Leaves of Arabidopsis Plants

SRMs on the TSQ Quantis Triple Quadrupole MS were used for analyzing metabolites of interest between wild-type Arabidopsis and a melatonin triple mutant. The targets were focused on metabolites related to the melatonin synthesis pathway and phytohormones. For each metabolite, two transitions were chosen based on the mzVault library (Supplemental Table S1). The targeted SRMs were run at a collision energy of 30 eV. The stacked ring ion guide was run using the tuned voltages of the Extended Range Mass Spectrometry Solution (Thermo Fisher Scientific, Rockford, IL, USA), and the capillary temperature was set to 320 °C. The resolution of both Q1 and Q3 was set to 0.7. The UHPLC system was the same as the one used for untargeted metabolomics. The raw data files were imported into the Thermo Xcalibur^TM^ software for the inspection of the metabolite peaks in the targeted scans using Qual Browser^TM^ and to quantify the areas of the peaks using Quan Browser^TM^ (Thermo Scientific, San Jose, CA, USA).

## 3. Results

### 3.1. mzVault Plant Metabolite Spectral Library

Based on the commercial availability of more than 2800 metabolites collected in the AraCyc database (https://pmn.plantcyc.org/ARA/class-tree?object=Compounds, accessed on 18 March 2022), 544 authentic standards were purchased and used for the creation of the mzVault spectral library. The criteria for inclusion in the current library were as follows: the compounds must weigh < 1500 Da, and they should be found at concentrations greater than 1 nM in Arabidopsis except for the low abundant but biologically important metabolites such as hormones and signaling molecules. A total of 510 metabolites were found in the KEGG database. Out of the 510 KEGG metabolites, 328 metabolites were mapped in metabolic pathways using the KEGG database (Figure S1).

**Figure 1:**
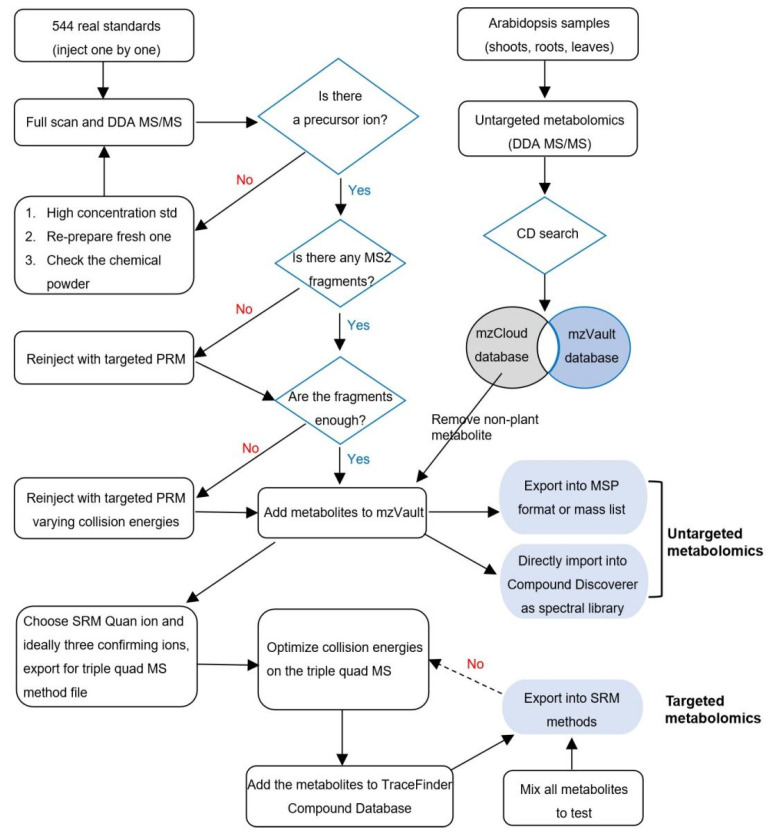
Workflow diagram showing the process of MS data collection and analysis of the 544 authentic standards, and the applications of the created mzVault spectral library in untargeted and targeted metabolomics of Arabidopsis seedlings.

**Figure 2:**
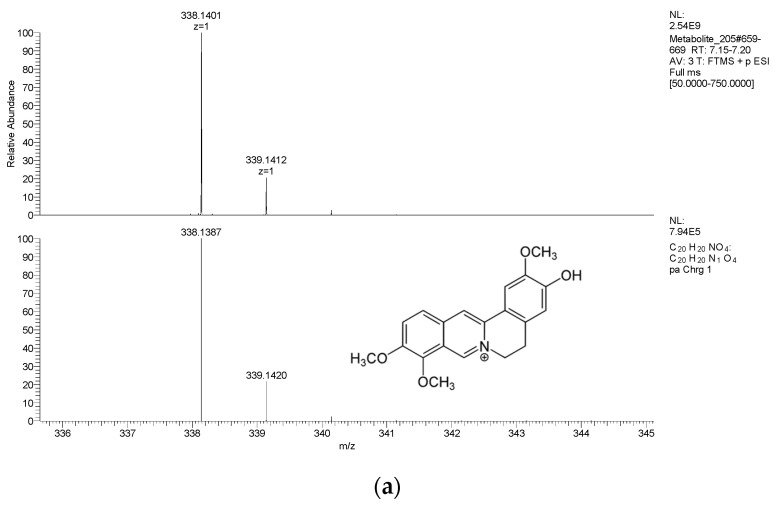

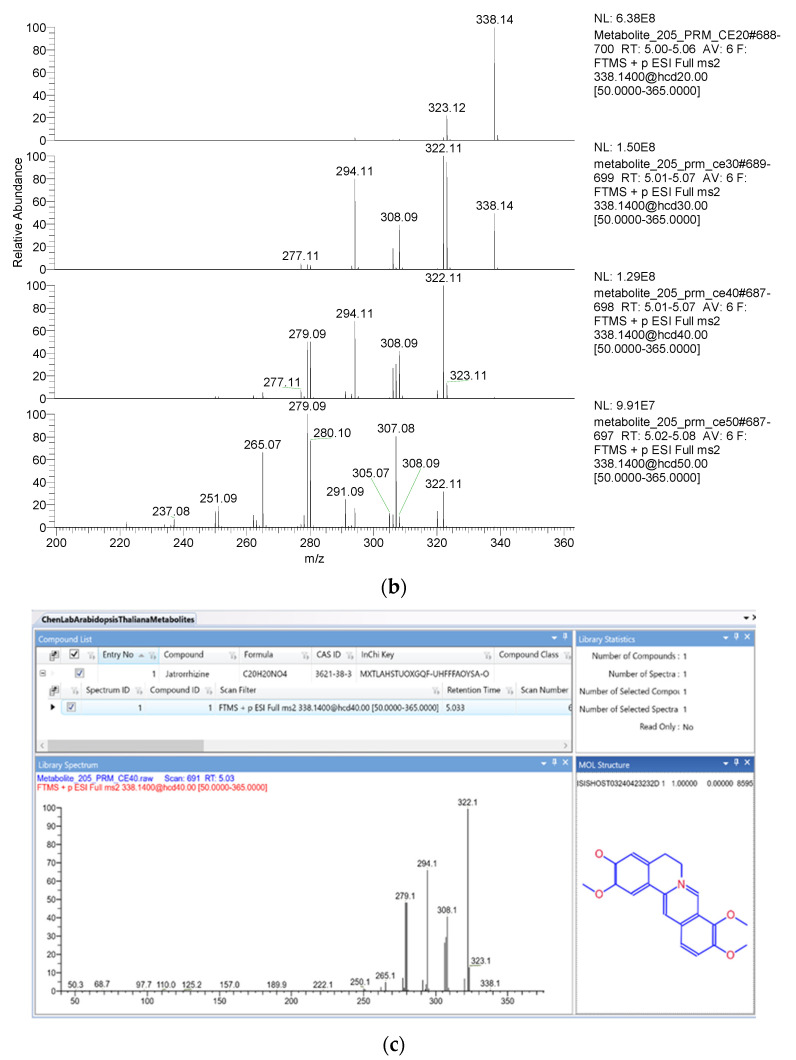
Full MS spectra and MS/MS spectra of metabolite 205 (jatrorrhizine) acquired at different normalized collision energy (NCE) values. (**a**) Full MS of jatrorrhizine. Top panel, experimental data; bottom panel, theoretical isotopic pattern. (**b**) MS/MS spectra of jatrorrhizine. The fragmentation spectra were acquired at NCEs of 20, 30, 40, and 50 eV from the top to bottom. (**c**) Entry of the jatrorrhizine spectra into the mzVault library (showing NCE 40 eV spectrum as an example).

Figure 1 shows the decision-tree diagram of the MS data acquisition and analysis of the 544 authentic standards. Both positive and negative full MS and data-dependent MS/MS were collected from the 544 authentic standards. Among them, 502 authentic standards had assignable precursor ions and MS/MS fragments. The other 42 compounds were excluded from the mzVault library. Here, metabolite 205, jatrorrhizine, was chosen to illustrate the criterion to assign a precursor ion and MS/MS ions (Figure 2). The full scan spectra appeared in both the positive and negative modes from the Q Exactive. The response in the positive mode was two orders of magnitude higher than in the negative mode. The experimental data are shown in the top panel, and the theoretical isotopic pattern is shown in the bottom panel (Figure 2a). The mass accuracy was less than 5 ppm and the isotopic abundances matched well. To optimize the fragmentation pattern, different NCEs were tested. At 20 eV, the intensity was great, but the MS/MS spectrum was dominated by the precursor ion. As NCE increased from 20 eV to 50 eV, more extensive fragmentation of the metabolite could be observed (Figure 2b). The ideal spectra showed a little precursor m/z with a balance of product ions across the mass range of the scan. With this data, an entry of jatrorrhizine was made in the mzVault, and spectra were saved into a local mzVault library (Figure 2c). Accordingly, all the spectra collected from the 502 metabolites with data-dependent MS/MS spectra from the Q-Exactive were added to the mzVault library.

**Figure 3:**
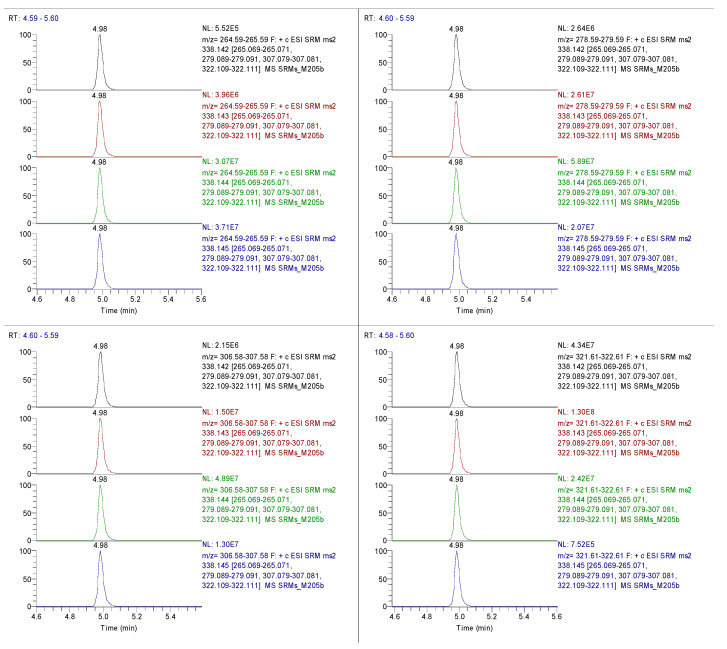
Establishing triple quadrupole SRMs with the optimized collision energies based on four large product ions of jatrorrhizine. The four panels represent collision energies of 20, 30, 40, and 50 eV, respectively.

Based on the MS/MS spectra (Figure 2), four product ions of jatrorrhizine with high intensities (m/z 265.07, 279.09, 307.08, and 322.11) were chosen as SRM transitions (Figure 3). In one method, each fragment ion was plotted in an Xcalibur Qual Browser to determine which energy gave the highest ion signal (chromatographic break-down curves). The optimal collision energies for the ions were 265.07 @ 50 eV, 279.09 @ 40 eV, 307.08 @ 40 eV, and 322.11 @ 30 eV. Then, all the transitions for the 502 metabolites were added to the TraceFinder Compound Database, and unscheduled SRMs were used to determine the chromatography and MS/MS performance of each metabolite in a mixture of metabolites.

### 3.2. Untargeted Metabolomics of Arabidopsis Leaves with the mzVault Spectral Library

mzCloud is one of the largest spectral libraries, with 19,699 compounds from authentic standards. However, for plant metabolites, the mzCloud database is limited, and there are many non-plant endogenous metabolites. To examine the utility of the created mzVault spectral library, the extracts from wild-type Arabidopsis leaves were used for untargeted metabolomics analyses. The obtained MS raw data files were submitted to Compound Discoverer for database searching using the Arabidopsis mzVault library created in this study and the mzCloud library. The identification is based on MS/MS spectral matching, which is deemed to be level two of metabolite identification [26].

**Figure 4:**
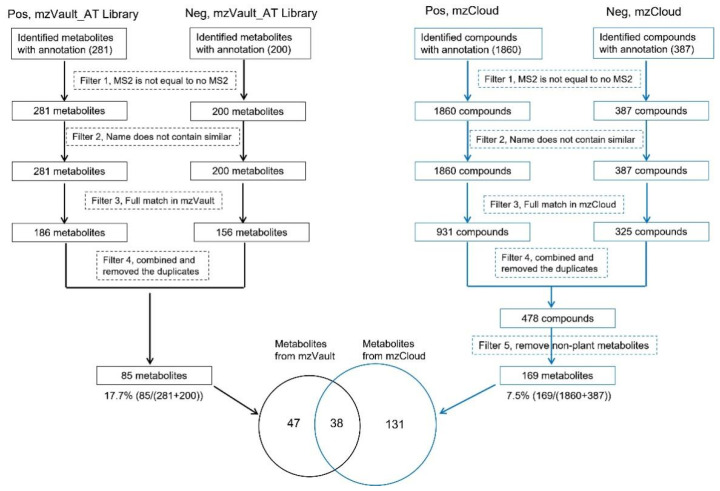
Comparison of the identified metabolites in untargeted metabolomics of Arabidopsis leaves using the created mzVault and the mzCloud libraries. The identification was based on the MS/MS spectral matching, i.e., at level two of the metabolite identification [26].

As shown in Figure 4, the numbers of identified metabolites with annotation and MS/MS spectra were 281 for the positive mode and 200 for the negative mode when the mzVault library was used. Where the mzCloud was used, there were 1860 and 387 identified metabolites for the positive and negative modes, respectively. After filtering the fully matched spectra in each library, 186 metabolites for the positive and 156 metabolites for the negative mode were obtained only using the mzVault, while 931 compounds for the positive and 325 compounds for the negative mode were obtained using mzCloud. Furthermore, after combining the metabolites or compounds identified in positive and negative modes and removing the redundant entries, a total of 85 metabolites from the mzVault library and 478 compounds from the mzCloud library were obtained. However, for the 478 compounds from the mzCloud library, we reviewed the compounds one by one to remove the non-plant-derived metabolites based on the literature, PubChem, and ChEBI databases. Only 169 metabolites from the mzCloud library were left (Figure 4). Clearly, the mzCloud library was not effective for plant metabolomic studies. The Venn diagram in Figure 4 shows that 38 metabolites were commonly identified between the mzVault and mzCloud libraries, and 47 metabolites were specially identified in the Arabidopsis mzVault library created in this study.

### 3.3. Targeted Metabolomics Enabled by the mzVault Plant Spectral Library

**Figure 5:**
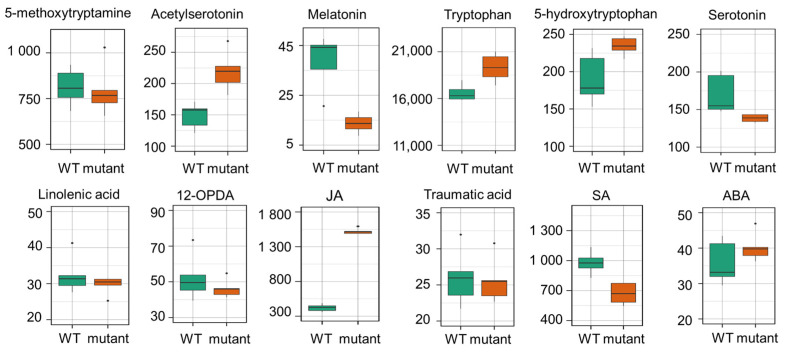
Relative quantification of melatonin synthesis-related metabolites (top six bar plots) and linolenic acid, 12-oxo Phytodienoic acid (OPDA), jasmonic acid (JA), traumatic acid, salicylic acid (SA), and abscisic acid (ABA) in wild type (WT) and a melatonin mutant. The y-axis represents peak areas in thousands.

Using the generated metabolite spectra, we were able to design SRM transitions for pseudo-targeted metabolomics on triple quadrupole instruments, as shown in Figure 3. With the MS/MS transitions of the metabolite of interest, obtaining authentic standards may not be necessary for relative quantification. As a proof-of-concept, 54 metabolites were analyzed for relative quantification using SRMs (Supplemental Table S2). As shown in Figure 5, the relative levels of 12 metabolites, including six melatonin pathway metabolites and six hormone-related metabolites (e.g., linolenic acid, 12OPDA, and traumatic acid), were determined in the wild type, and a melatonin biosynthesis mutant, *asmt1snat1comt1*. Although the melatonin biosynthesis pathway in plants has not been fully revealed yet, studies have shown that ASMT1, COMT1, and SNAT1 play roles in the melatonin biosynthesis of Arabidopsis [20,27,28,29]. As expected, the triple mutant has significantly reduced levels of melatonin, indicating the indispensable roles of ASMT1, COMT1, and SNAT1 in the melatonin biosynthesis of Arabidopsis. Other melatonin biosynthesis intermediates such as N-acetylserotonin, 5-hydroxytryptophan, serotonin, and 5-methoxytryptamine were either slightly increased or unaltered in their abundance. Considering that plant melatonin biosynthesis occurs through at least two different routes, unlike animals [30], further metabolite analysis with their single and double mutants would help to elucidate melatonin biosynthesis. Interestingly, JA was elevated, and SA decreased in the mutant compared to the wild type (Figure 5). Although the biological implications are not known, the results showed the utility of the spectral library-assisted targeted SRMs in generating interesting testable hypotheses.

## 4. Discussion

### 4.1. Data Acquisition for Plant Metabolite Spectral Library

With the development of high-throughput mass spectrometry technology, MS-based metabolomics has become a common approach for metabolite identification using public spectral libraries and databases [31]. Currently, one of the major limitations of plant metabolomics is the MS spectral annotation and, thereby, chemical structure identification. Unlike human metabolome databases, there are very few plant-specialized spectral libraries based on authentic standards for plant metabolomics. Although several MS/MS databases have been established for metabolite annotation, such as mzCloud, MassBank, and METLIN [32], major challenges for plant metabolomics include the low representation of plant metabolites and misidentification with non-plant metabolites, as well as a barrier to full access (e.g., free download of the database file and import into software programs). In this study, a non-redundant plant metabolite spectral library was established using authentic plant metabolite standards. It is publicly available and can be downloaded from the Zenodo as a mzVault database file for Compound Discoverer searching or in MSP format or mass list for another software searching. In addition, the mzVault platform allows the community to expand and improve the spectral library in the future. Furthermore, the MS/MS data at different collision energies for each metabolite greatly enhanced its usability for analysis of metabolic data acquired on different instruments.

### 4.2. Design of SRM Transitions from the mzVault Library

SRM is a common LC-MS/MS method for targeted proteomics and metabolomics [33]. Traditionally, the SRM method’s development depended on the availability of authentic standards. It is expensive to obtain a large number of chemical standards. Based on the high-resolution Orbitrap data, the sequentially stepped targeted MS/MS (sst-MS/MS) method was developed for targeted peptide analysis. After acquiring global comparative proteomics data using Q-Exactive Plus mass spectrometer, 32 changed proteins were validated using the SRM of selected peptides that were unique to those proteins [34]. Similarly, Gu et al. used globally optimized targeted MS for metabolomics that combined the advantages of targeted detection and untargeted profiling with an LC−triple quadrupole MS [35]. Recently, untargeted metabolomic data obtained on a Q-TOF MS/MS was used to design 518 SRM ion pairs for quantifying hundreds of metabolites in real hepatocellular carcinoma samples [36]. In this study, SRM transitions were designed based on the spectral library. The SRM method was exported from the TraceFinder compound database for use as targeted metabolomics on triple quadrupole MS. Cleary, the mzVault spectral library enables pseudo-targeted metabolomics without the need of obtaining authentic standards. Importantly, our mzVault spectral library can be easily expanded and incorporated into the libraries with the freely available mzVault package.

### 4.3. mzVault Spectral Library Improved Metabolite Identification in Untargeted Metabolomics

LC-MS/MS-based untargeted metabolomics profile hundreds of metabolites in plant cells [37,38]. However, in contrast to highly reproducible GC-MS spectra (based on a commonly accepted electron energy), the MS/MS spectra produced from different LC-MS/MS platforms were highly variable (there was no consensus on gas pressure, gas composition, collision energy, or reference spectra). This has limited metabolite annotation in untargeted metabolomics [39]. Although LC-MS/MS platforms have advanced in recent years, no one platform or instrument can achieve comprehensive untargeted metabolomics [40,41]. To overcome this challenge, we created this plant-specific mzVault library, where each compound has multiple spectra at different NCEs. Using this specific metabolite library, more metabolites were identified than just using the mzCloud (Figure 4). Considering the total sizes of the two libraries, it becomes obvious that the mzVault is more effective. Different from other databases, mzVault is plant-specific, and one compound contains various NCEs in the mzVault (Figure 2), which maximizes the probability of obtaining informative matching of the MS/MS spectra for level two metabolite identification. In mammalian liposomes, the application of multiple NCEs has been described [42]. Here, in addition to NCEs, accurate measurements of the precursor m/z, retention time (RT), and fragmentation spectra of each metabolite were established in the mzVault library. These parameters enabled dependable identifications of plant metabolites in untargeted plant metabolomics (Figure 4). However, compared to the more than 200,000 metabolites which the plants produce [43], only a relatively small number of metabolites were commercially available for building the MS spectral libraries [39,44]. The current spectral library and additional information about known metabolic reactions/transitions and physiological concentrations, etc., in PubChem, Chemspider, KEGG, and HMDB, may assist artificial intelligence efforts toward the accurate prediction of metabolite spectra similar as what has been achieved with peptides [45,46] and protein structures [47].

### 4.4. mzVault Enabled Hyphenated Targeted and Untargeted Metabolomics

For comprehensive metabolomics, a combination of untargeted and targeted metabolomics was developed to enhance the metabolome coverage. Based on the circulating metabolites of rats, the model of cardiac arrest and cardiopulmonary resuscitation was identified with the combination of untargeted and targeted metabolomics [48]. In plants, the combined metabolomics was adopted to reveal time-resolved metabolomics, which changed under elevated CO_2_ [49]_._ Additionally, untargeted metabolomics generated discovery data that may be validated through hypothesis testing using targeted metabolomics with SRM [50]. However, the classic SRM assay was limited by the availability of authentic compound standards. With the development of the mzVault spectra library, relative quantification using the targeted SRM assay is feasible without authentic standards. Importantly, the mzVault MS/MS spectral library enabled hyphenated untargeted and targeted metabolomics for the broad coverage of plant metabolomes.

## 5. Conclusions

In this study, we have created an MS1 and MS2 spectral library of 502 plant metabolites. The benefits of this unique plant metabolite spectral library are sevenfold. First, the MS1 and MS2 spectra were acquired at high resolutions and with high mass accuracy (<5 ppm). Additionally, the MS2 spectra for each metabolite were generated at different collision energies. This ensures the high success of metabolite identification based on the spectral matching of data generated on different instrument platforms (a user could choose one or more compounds from the library and vary gas pressure or collision energy to match the library). Second, as shown in our proof-of-concept applications, this spectral library is useful for untargeted plant metabolomics and targeted metabolomics. It is obvious that this spectral library has increased the number of chemically identified metabolites in complex samples. It has also allowed SRM-based targeted metabolomics without the need for authentic standards. Third, since mzVault is freely downloadable, this spectral library can be enlarged with the contribution of the community as soon as more authentic compounds become available. Clearly, this mzVault spectral library is an important resource for the community to springboard plant metabolomics research and development. Last but not least, the approach of establishing the mzVault libraries is broadly applicable to many other organisms. The established spectral library may be used as a training set together with other public libraries and databases toward the artificial intelligence prediction of the MSn spectra of metabolites without authentic standards.

## Data Availability

The mzVault spectral library is available through Zenodo repository DOI: 10.5281/zenodo.6916522 and GNPS repository DOI: 10.25345/C5RX93J4D.

## Supplementary Materials

The following supporting information can be downloaded at: https://www.mdpi.com/article/10.3390/ijms24032249/s1.
