# Supplementary material for: Creation of a Plant Metabolite Spectral Library for Untargeted and Targeted Metabolomics
Source: Int J Mol Sci. 2023 Jan 23;24(3):2249. doi: 10.3390/ijms24032249 (PMC9916794; doi:10.3390/ijms24032249)
Supplement: Supplementary file 1 [file ijms-24-02249-s001.zip › supplementary Figure S1.pdf]

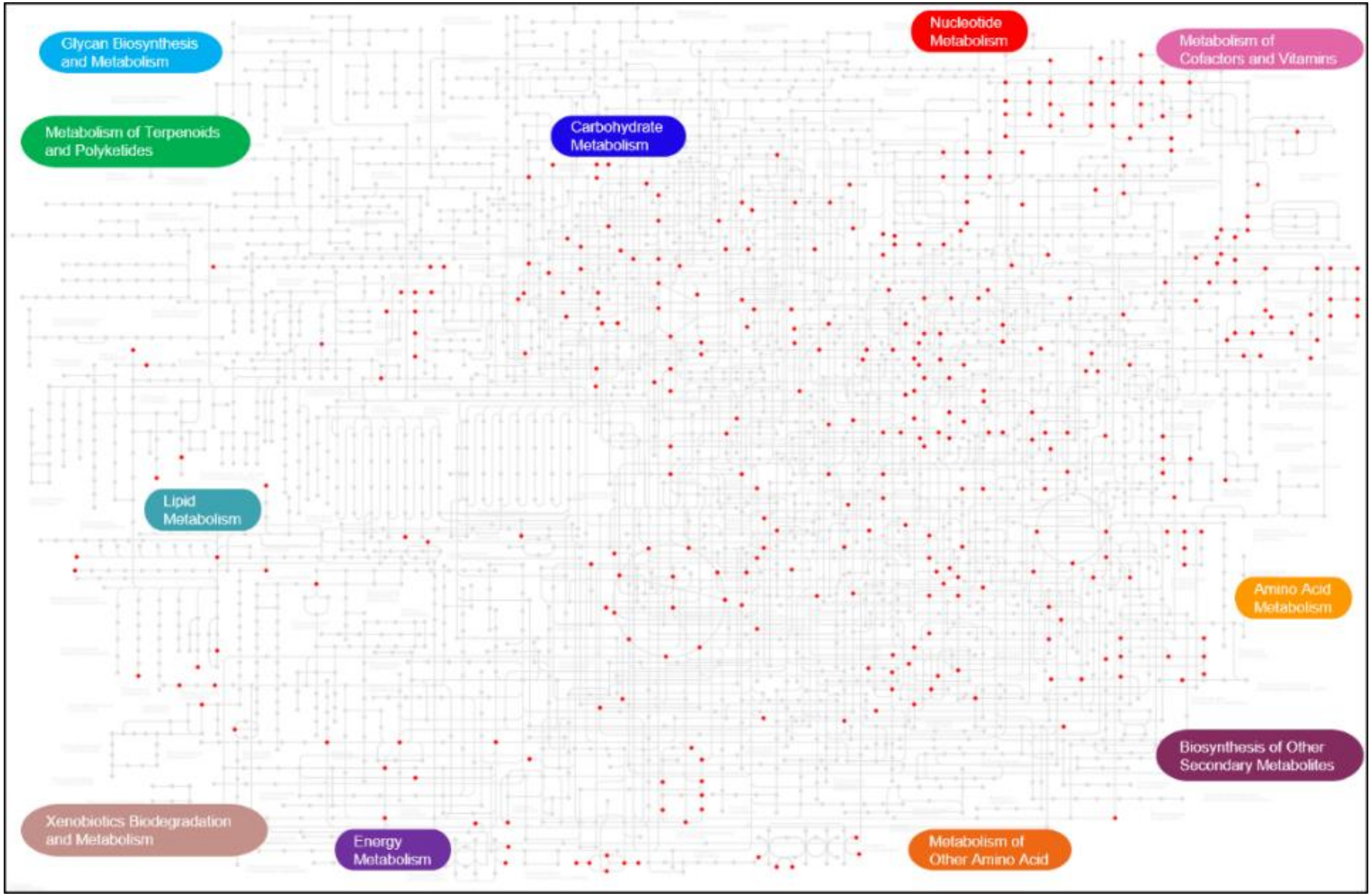

Figure S1. KEGG pathway map coverage of the metabolites in the mzValut library. The red dots indicate the mapped metabolites.
